# Evaluation of the Swelling Properties and Sorption Capacity of Maltodextrin-Based Cross-Linked Polymers

**DOI:** 10.3390/gels10040232

**Published:** 2024-03-28

**Authors:** Claudio Cecone, Gjylije Hoti, Fabrizio Caldera, Marco Ginepro, Adrián Matencio, Francesco Trotta

**Affiliations:** 1 Department of Chemistry, Nanomaterials for Industry and Sustainability Centre (NIS Centre), Università degli Studi di Torino, Via P. Giuria 7, 10125 Turin, Italy; gjylije.hoti@unito.it (G.H.); fabrizio.caldera@unito.it (F.C.); marco.ginepro@unito.it (M.G.); adrian.matencioduran@unito.it (A.M.); francesco.trotta@unito.it (F.T.); 2 Department of Drug Science and Technology, Università degli Studi di Torino, Via P. Giuria 9, 10125 Turin, Italy

**Keywords:** maltodextrin-based gels, cross-linking density, sorbents, water remediation, green processes

## Abstract

The development of polymers obtained from renewable sources such as polysaccharides has gained scientific and industrial attention. Cross-linked bio-derived cationic polymers were synthesized via a sustainable approach exploiting a commercial maltodextrin product, namely, Glucidex 2^®^, as the building block, while diglycidyl ethers and triglycidyl ethers were used as the cross-linking agents. The polymer products were characterized via FTIR-ATR, TGA, DSC, XRD, SEM, elemental analysis, and zeta-potential measurements, to investigate their composition, structure, and properties. Polydispersed amorphous granules displaying thermal stabilities higher than 250 °C, nitrogen contents ranging from 0.8 wt % and 1.1 wt %, and zeta potential values between 10 mV and 15 mV were observed. Subsequently, water absorption capacity measurements ranging from 800% to 1500%, cross-linking density determination, and rheological evaluations demonstrated the promising gel-forming properties of the studied systems. Finally, nitrate, sulfate, and phosphate removal tests were performed to assess the possibility of employing the studied polymer products as suitable sorbents for water remediation. The results obtained from the ion chromatography technique showed high sorption rates, with 80% of nitrates, over 90% of sulfates, and total phosphates removal.

## 1. Introduction

Maltodextrins are water-soluble D-glucose oligomers that are widely employed in the food and pharma industry [1,2]. They can be obtained from starch via enzymatical or thermal treatments. Maltodextrins are characterized by specific dextrose equivalent (DE) values that are usually lower than 20. This value represents the reducing equivalent of maltodextrin against the same mass of glucose. Usually, the higher the dextrose equivalent, the lower the molecular weight of the maltodextrin [3,4]. Because of maltodextrins’ good water solubility, numerous studies reported the possibility of cross-linking them into insoluble polymer networks by exploiting suitable cross-linkers, aiming for application in aqueous media [5,6,7,8,9,10]. However, many of these compounds, namely, epichlorohydrin, diphenyl carbonate, and isocyanates, are toxic and require the use of organic solvents. As lower-toxicity alternatives, water-soluble diglycidyl ethers and triglycidyl ethers have been studied and reported [11,12,13,14,15,16,17]. 1,4 butanediol diglycidyl ether has been reported by Martucci et al. [18] as a suitable cross-linker for the preparation of bovine gelatin-based films for packaging applications. Flexible soy-based adhesives, which are suitable for producing plywood, were obtained by Luo et al. [19] exploiting neopentyl diglycidyl ether, while trimethylolpropane triglycidyl ether was used to obtain a vanillin-derived vitrimer by Roig et al. [20]. Polymer hydrogels displaying a three-dimensional polymer network and high solvent absorption capacities have shown promising performance if employed as sorbents for dyes, heavy metal ions, and other organic hazardous pollutants [21,22,23]. This is mainly related to their swelling properties, which are associated with different possible sorption routes. Bio-derived polymer hydrogels have various advantages such as sustainability and the possibility of displaying favorable mechanical properties that make them suitable candidates for the removal of potential pollutants [24,25,26].

The presence of nitrogen and phosphorus, and the toxicity associated with nitrates, are considered the main cause of the degradation of rivers, lakes, and marine compartments around the planet [27,28,29,30,31]. In recent decades, a significant increase in nitrate concentrations in surface water, groundwater, and agricultural areas associated with intensive livestock activities has been reported [32]. Furthermore, the use of fertilizers and plant protection products has been determined to have a significant impact on the territory and water resources [33,34]. In addition, high contents of sulfates due to natural soil composition, industrial processes, or atmosphere emissions represent a threat to the ecosystem, while phosphates are often the main cause of a poor-quality state of groundwaters that are, by far, the primary source of drinking water production [35,36,37,38,39,40,41]. Despite the development of specific water treatments, e.g., chemical denitrification, electrodialysis, reverse osmosis, and nanofiltration, the new technologies necessary to meet more strict environmental requirements are still being widely investigated [42,43]. Bio-derived materials are more sustainable than conventional petrochemical ones. Consequently, the development of sorbents obtained from renewable sources such as polysaccharides has gained scientific and industrial attention, this being also in harmony with the aims of green chemistry [44,45,46,47,48].

In this work, the synthesis of maltodextrin-based cross-linked polymers was optimized via a sustainable approach by using 1,4 butanediol diglycidyl ether and trimethylolpropane triglycidyl ether as cross-linking agents. Afterward, the products were characterized via Fourier transform infrared spectroscopy, thermogravimetric analysis, differential scanning calorimetry, X-ray diffraction, elemental analysis, and zeta potential. The gel-forming characteristics were subsequently investigated via water absorption capacity measurements, cross-linking density determination via the Flory–Rehner theory, and rheological behavior. Lastly, the polymers were tested as suitable sorbents for the sorption of nitrates, sulfates, and phosphates from water.

## 2. Results and Discussion

### 2.1. Material Design and Characterization

**Figure 1:**
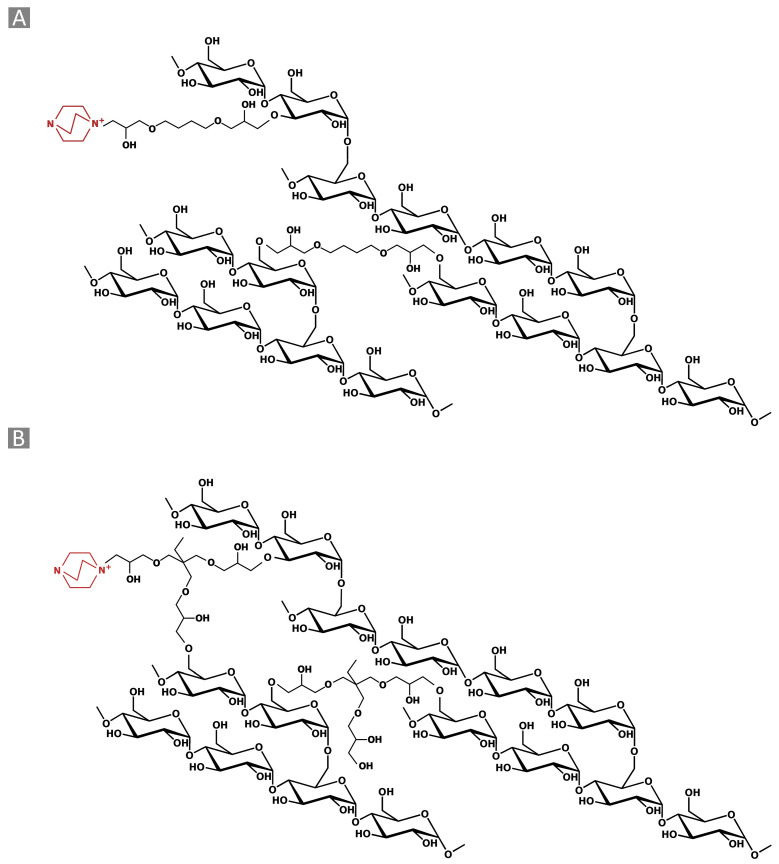
Schematic representation of (**A**) GLU_BDE and (**B**) GLU_TTE.

**Figure 2:**
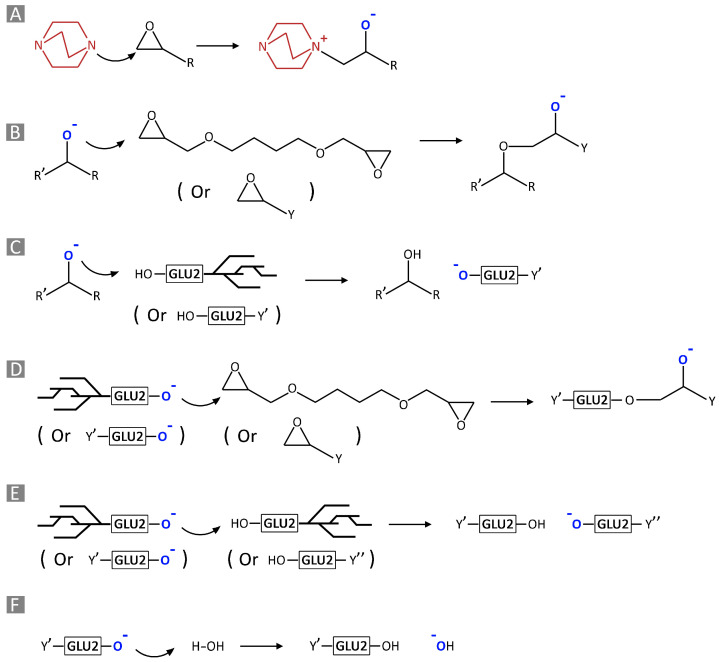
Amine-mediated ring-opening reactions (**A**). Chain growth reactions by alkoxide species (**B**–**E**); the termination reaction (**F**).

Aiming to obtain bio-derived water-insoluble polymers, the amine-mediated ring-opening reaction of di-glycidyl (1,4 butanediol diglycidyl ether, BDE) and tri-glycidyl (trimethylolpropane triglycidyl ether, TTE) ethers has been exploited to cross-link a commercial maltodextrin (Glucidex 2^®^, GLU2), yielding cationic polymer structures as the product (Figure 1). An initial interaction, occurring between 1,4-diazabicyclo [2.2.2] octane (DABCO) and the cross-linker (Figure 2A), was reported to generate alkoxide species, which subsequently led to the growth and end of polymer chains (Figure 2B–F) [26]. Once formed, these alkoxides can react with either other epoxide rings belonging to the linker or hydroxyl functions from GLU2. In the first case, the reaction is mostly associated with the propagation of the alkoxide species, along with a negligible growth in molecular weight. Conversely, the alkoxides generated on the backbone of GLU2 molecules, which can further react with other epoxide rings, are considered to have a great impact on increasing the molecular weight.

**Figure 3:**
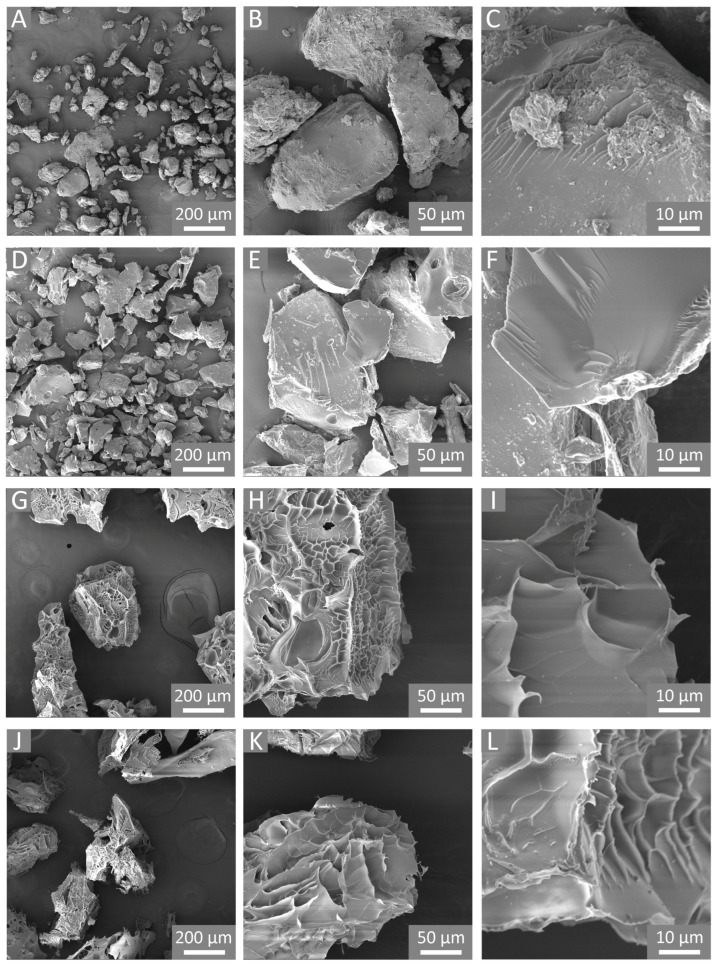
SEM images of (**A**–**C**) GLU_BDE, (**D**–**F**) GLU_TTE, and (**G**–**I**) GLU_BDE after swelling, and (**J**–**L**) GLU_TTE after swelling.

As a result of the above-described reactive path, cationic cross-linked polymers were obtained. Additionally, owing to the atom economy of the epoxide ring-opening reactions, excellent yields were observed for all the polymers. For each performed synthesis, the mass balance was calculated as the mass of the final product after purification and drying versus the theoretical mass, equal to the sum of the masses of GLU2, cross-linker, and DABCO. A mass balance of 88% and 85% was observed for GLU_TTE and GLU_BDE, respectively. As displayed in Figure 3A–F, the polymer products were characterized by polydisperse granules with a size of between approximately a few tens and two hundred microns. The morphological characterization also revealed the presence of smooth external surfaces where no macroporosity or mesoporosity was detected.

**Figure 4:**
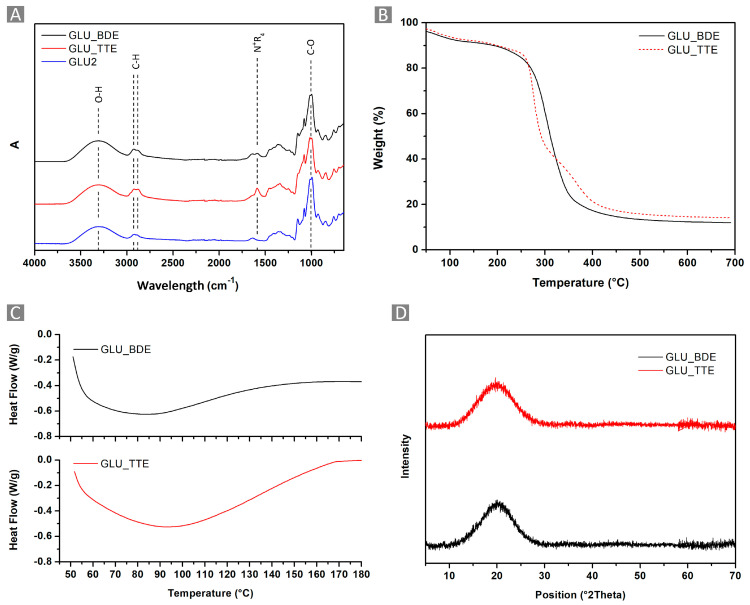
(**A**) FTIR-ATR spectra, (**B**) TGA, (**C**) DSC, and (**D**) XRD of GLU_BDE and GLU_TTE.

From the FTIR-ATR analysis of the polymers reported in Figure 4A, the typical IR signals of the maltodextrin and its cross-linkers were observed. The bands at 2921 cm^−1^ and 2867 cm^−1^ were related to C–H stretching modes, while the vibrations of the glucopyranose cycle and C–H bond deformation were related to the regions at 950–650 cm^−1^ and 1400–1150 cm^−1^. The spectral region between 3000 cm^−1^ and 3500 cm^−1^ was characterized by a large band related to the symmetric and anti-symmetric O–H stretching modes, whereas at 1645 cm^−1^, the OH bending signals were detected. The C-O-C or C-O bond vibrations of both maltodextrin and cross-linkers were visible in the region of 1080–1000 cm^−1^. Eventually, the occurrence of amino-mediated ring-opening reactions, resulting in quaternary ammonium functions, was detected as a shoulder at 1590 cm^−1^.

The thermograms reported in Figure 4B were characterized by a first weight loss occurring up to approximately 150 °C, which was related to the volatilization of the water adsorbed on the samples. Subsequently, between 250 °C and 450 °C, a single-step decomposition process was observed for GLU_BDE, whereas the GLU_TTE profile indicated a multistep decomposition process. Additionally, a stable carbon residue up to 700 °C was obtained, corresponding to roughly 20% of the initial weight. Also, GLU_BDE displayed a higher T_onset_ equal to 271 °C, in comparison with GLU_TTE, which displayed a T_onset_ equal to 264 °C. The DSC and XRD reported in Figure 4C and Figure 4D, respectively, indicate how GLU_BDE and GLU_TTE are characterized by amorphous polymer networks, as reasonably correlate with cross-linked macromolecular structures.

The presence of nitrogen atoms composing the polymer structure, associated with positively charged pendants, was further confirmed by the elemental analysis of the samples. A nitrogen content of 1.1 wt % was detected in GLU_TTE and one of 0.8 wt % in GLU_BDE. Furthermore, the nitrogen quantification obtained via elemental analysis was supported by zeta-potential measurements. GLU_TTE displayed a zeta-potential equal to 13.8 ± 1.7 mV, which was related to a higher presence of nitrogen in comparison with GLU_BDE, which displayed a zeta-potential of 10.4 ± 1.2 mV. The observations so far showed how the presence of tri-functional cross-linkers such as TTE, compared to di-functional ones like BDE, are associated with products bearing higher cationic features. This aspect has been related to the capability of tri-functional cross-linkers to act both as a cross-linker between two GLU2 molecules and as a cationic pendant by reacting with DABCO, exploiting the remaining epoxy function.

### 2.2. Water Absorption Capacity

**Figure 5:**
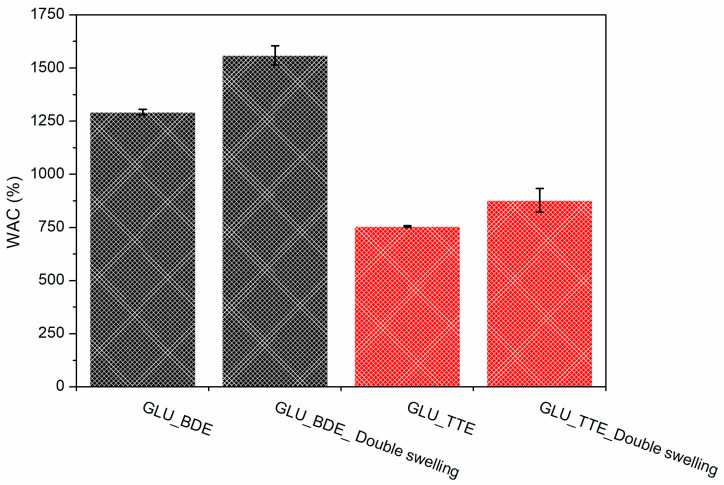
WACs of GLU_BDE and GLU_TTE. The WAC of GLU_BDE after swelling and subsequent freeze-drying (GLU_BDE_Double swelling), and GLU_TTE after swelling and subsequent freeze-drying (GLU_TTE_Double swelling).

The water absorption capacity (WAC) of a polymer network affects the diffusion of solutes into and out of a swollen polymer matrix and the latter’s mechanical properties; therefore, it is an important aspect worth studying. The synthesized GLU_BDE and GLU_TTE were placed in water and allowed to swell until equilibrium was reached. The WAC values, as presented in Figure 5, ranged from 755 ± 5% for GLU_TTE to 1293 ± 13% for GLU_BDE. The WAC was higher when GLU2 was crosslinked with a di-functional cross-linker (BDE) than with a tri-functional one (TTE). This aspect is likely associated with a more compact and dense structure in the case of GLU_TTE, resulting in a lower availability to absorb water. Negligible differences in the WAC for both GLU_BDE and GLU_TTE were obtained by performing the tests at pH values ranging from 5 to 9. The swollen GLU_TTE and GLU_BDE were subsequently freeze-dried, and their WAC was further evaluated. The thus-formed gels show a slightly superior WAC, as reported in Figure 5. A WAC of 878 ± 56% was observed for GLU_TTE, while 1560 ± 46% was obtained for GLU_BDE. This might indicate rearrangement of the polymer network taking place while the polymers are in a swollen state and then subjected to a subsequent freeze-drying process.

The SEM images of freeze-dried GLU_BDE (Figure 3G–I) and freeze-dried GLU_TTE (Figure 3J–L) show a different morphology in comparison with the same polymers before the swelling and subsequent freeze-drying processes. In the literature, it has been proposed that due to the freezing of water and sublimation of ice crystals, the polysaccharide chains can be subjected to modifications and rearrangement [49]. Presumably, the growth of ice crystals through the freezing of free water led to the stretching of the macromolecular network and the subsequent deformation of the polymer matrix. Also, the ice crystals being formed without any specific orientation led to negligible differences between the two freeze-dried polymers. The resulting irregular and heterogeneous surfaces are reasonably related to the shrinking of cross-linked swollen systems to a dry state.

### 2.3. Cross-Linking Density Determination Using the Flory–Rehner Theory

**Figure 6:**
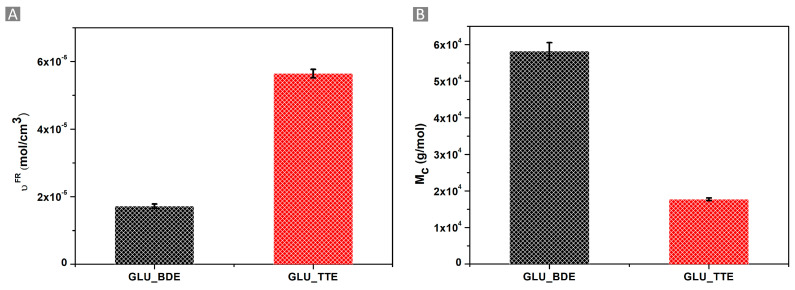
(**A**) Cross-linking density, as determined by the Flory–Rehner theory (υ^FR^), and (**B**) the molecular weight between two cross-links (M_c_) for GLU_BDE and GLU_TTE.

**Figure 7:**
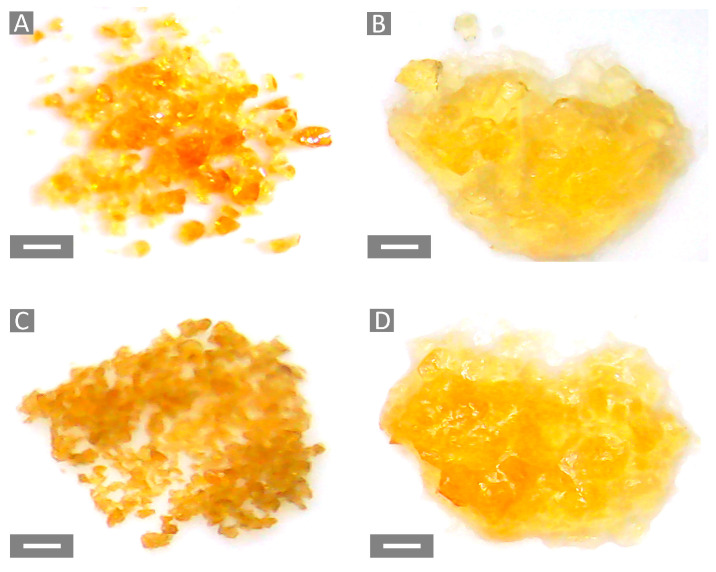
Images of (**A**) GLU_BDE and (**C**) GLU_TTE in a dry state. (**B**) GLU_BDE and (**D**) GLU_TTE in a swollen state. Scale bar: 1 mm.

The characterization of a gel is a complex procedure wherein the cross-linking density of the system determines the mechanism of water diffusion into the polymer network [50]. Despite the complexity of the network structure, the Flory–Rehner theory can lead to the calculation of the average values for cross-linking density (υ^FR^) and the molecular weight between two cross-links (M_c_). The high WAC observed for GLU_BDE resulted in a lower cross-linking density equal to 1.72 × 10^−5^ ± 6.55 × 10^−7^ mol/cm^3^, whereas a higher cross-linking density of 5.64 × 10^−5^ ± 1.26 × 10^−6^ mol/cm^3^, associated with a lower WAC, was obtained for GLU_TTE, as reported in Figure 6A. A higher WAC was related to the increased free volume available in the polymer network, i.e., the distance between two cross-linking points. GLU_BDE was characterized by a higher M_c_ of 58,212 ± 2322 g/mol, if compared with GLU_TTE which exhibited 17,724 ± 385 g/mol (Figure 6B). When the number of crosslinks per mass unit is increased, a more densely cross-linked network can be observed, which is associated with lower M_c_ values. Consequently, the systems can accommodate a lower amount of solvent. The investigation of the gels via an optical microscope (Figure 7) demonstrated that swollen GLU_TTE and GLU_BDE did not display noteworthy differences in terms of color and texture.

### 2.4. Rheological Measurements

**Figure 8:**
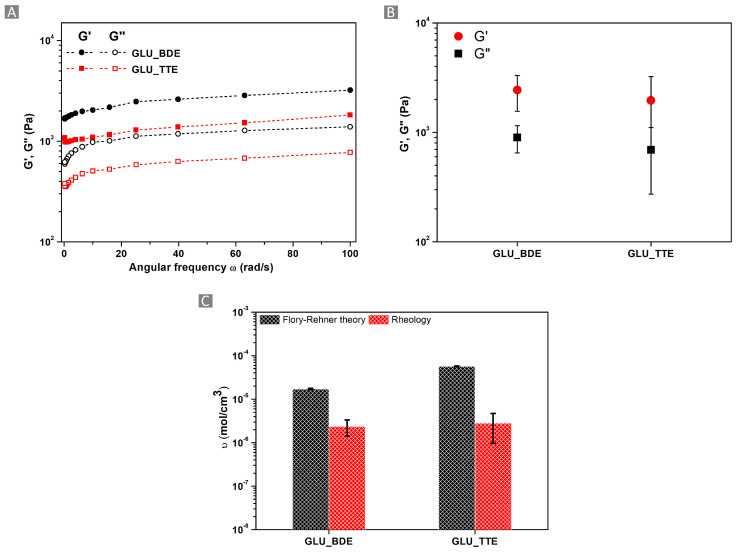
(**A**) G′ and G″ vs. angular frequency for GLU_TTE and GLU_BDE, with a 2 mm gap size. (**B**) G′ and G″ of GLU_TTE and GLU_BDE at ω of 1 rad/s, with a 2 mm gap size. (**C**) Comparison of cross-linking density (υ) values obtained with the Flory–Rehner theory and rheology.

The WAC is a crucial property when determining the mechanical stiffness of gels, which is mainly investigated by rheological measurement. Rheological properties were evaluated in isothermal conditions, specifically 25 °C, using a rheometer. A stress sweep test on a parallel plate rheometer under controlled strain conditions was performed to study the viscoelastic behavior of GLU_BDE and GLU_TTE. The storage modulus and loss modulus, known as G′ and G″, represent behaviors associated with viscoelastic solids and viscoelastic liquids, respectively. A storage modulus higher than the loss modulus was detected for all studied angular frequencies, acting as a confirmation of the gel-like behavior of GLU_BDE and GLU_TTE (Figure 8A). Also, the resulting storage modulus of GLU_BDE at an angular frequency of 1 rad/s, equal to 2435 Pa, was higher than the 1957 Pa value of GLU_TTE (Figure 8B). Subsequently, the storage modulus was used to calculate the density of polymer chains per volume unit (ʋ_e_^R^), according to Equation (4). Higher ʋ_e_^R^ values were observed for GLU_TTE in comparison with GLU_BDE, with values equal to 2.83 × 10^−6^ ± 1.86 × 10^−6^ mol/cm^3^ and 2.38 × 10^−6^ ± 9.68 × 10^−7^ mol/cm^3^, respectively, which is in agreement with the results obtained from WAC and cross-linking density via the Flory–Rehner theory. This aspect can be explained by the presence, in the case of GLU_TTE, of a more cross-linked and compact network that is favored by employing a tri-functional cross-linker during the polymer synthesis. In addition, a comparison between the cross-linking densities derived from the Flory–Rehner theory and the rheological measurements is depicted in Figure 8C. The two sets of cross-linking density values are consistent with each other, considering the separate approaches utilized for their determination.

### 2.5. Evaluation of Sorption Performances

**Figure 9:**
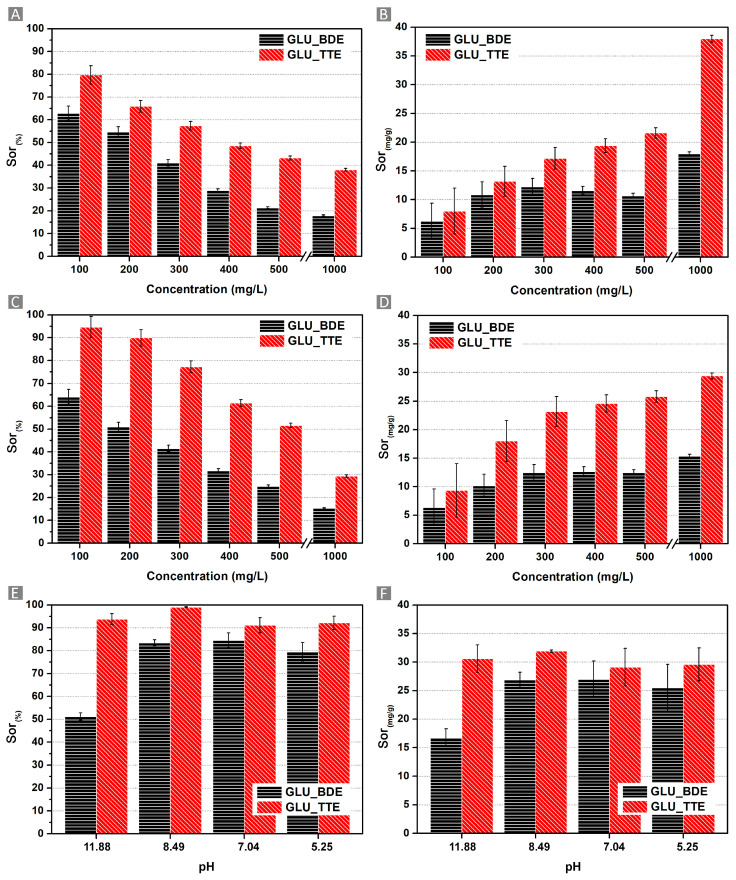
Results of the (**A**,**B**) nitrate sorption, (**C**,**D**) sulfate sorption, and (**E**,**F**) phosphate sorption tests. The first column shows the Sor_(%)_ value, while the second column reports the Sor_(mg/g)_ value.

The above-mentioned characterizations proved the good hydrophilicity and the presence of cationic sites of both GLU_BDE and GLU_TTE, which were subsequently screened as suitable sorbents for the removal of inorganic anions. The latter feature was hypothesized to be pivotal in the formation of electrostatic interactions between the polymers and the anions, increasing their removal from water. Therefore, the sorption features against nitrates, sulfates, and phosphates at various concentrations and pH values were explored. The sorption tests were performed for 100, 200, 300, 400, 500, and 1000 mg/L solutions in the case of nitrates and sulfates, while in the case of phosphates, the sorption tests were carried out at pH values of 11.88, 8.49, 7.04, and 5.25, keeping constant the concentration of phosphorous at 32 mg/L. In the case of nitrates (Figure 9A,B), the Sor_(%)_ changed with the variation of the initial concentration of the pollutant. GLU_TTE displayed higher Sor_(%)_ in comparison with GLU_BDE at all nitrate concentrations screened. When looking at Figure 9A, an overall decrease in the percentage of removal efficiency as the nitrate concentration increases can be observed. The reason behind this trend was hypothesized to be related to the number of cationic sites displayed by the sorbent, which can electrostatically interact with the pollutant, resulting in the retention of the latter. Once these sites are saturated by the presence of the corresponding moles of anions, the sorption capacity of the sorbent will drop, causing an overall decrease in the sorption performance at increasing pollutant concentrations. All the sorbents displayed the higher Sor_(%)_ from 100 mg/L solutions, where the highest value was observed for GLU_TTE, corresponding to 79.7 ± 4.0%, followed by GLU_BDE, with a Sor_(%)_ value of 62.9 ± 3.1%. Considering both the equilibrium characterizing the electrostatic interactions and the swelling capability of the polymer matrix, an increasing Sor_(mg/g)_ value was expected with a higher concentration of anions. In confirmation of this hypothesis, the Sor_(mg/g)_ increased proportionally to the concentrations of pollutants, as reported in Figure 9B. In this regard, a higher Sor_(mg/g)_ value was observed from 1000 mg/L solutions. GLU_TTE was the most high-performing system with 38.0 ± 0.6 mg/g, followed by GLU_BDE with 18.0 ± 0.3 mg/g. A similar trend with higher sorption capacities than nitrates at low concentrations was displayed in the sulfate sorption tests (Figure 9C,D). This result demonstrates how the sorption mechanisms are affected by the charge and dimensions displayed by each anion. GLU_TTE performed significantly better with a Sor_(%)_ value of 94.6 ± 4.7%, whereas GLU_BDE exhibited a Sor_(%)_ value of 64.2 ± 3.2%. What was observed was related firstly to the higher charge of sulfates if compared to nitrates, allowing the first to be more prone to electrostatically interacting with the sorbent. Also, sulfates are larger anions than nitrates, leading to their increased retention within the polymer network. When looking at the Sor_(mg/g)_ from 1000 mg/L solution, the performances decreased compared to the test carried out with nitrates for both GLU_TTE and GLU_BDE, with values corresponding to 15.4 ± 0.3 mg/g and 29.4 ± 0.5 mg/g, respectively. When considering the electrostatic interactions as the main phenomena behind the sorption process, the lower amount of sulfate sorbed than that of nitrate, was related to the higher number of sulfate charges, which saturated the active sites of the sorbent more rapidly.

Lastly, GLU_BDE and GLU_TTE were tested against four phosphate solutions at pH 11.88, pH 8.49, pH 7.04, and pH 5.25 (Figure 9E,F) to assess the pH dependency displayed by phosphates. Between pH 4 and pH 6, phosphates are mostly found as di-hydrogen phosphate species, and between pH 8 and pH 11, the mono-hydrogen phosphate is the predominant form, while above pH 12.5, the orthophosphate anion becomes the main form [51,52]. Having said this, the best removal performances were observed at pH 8.49, with a Sor_(%)_ value of 99.0 ± 0.2% for GLU_TTE and 83.5 ± 1.3% for GLU_BDE. These results are likely related to the higher number of charges of the orthophosphate than mono-hydrogen phosphate and di-hydrogen phosphate. Moreover, with increasingly alkaline conditions, the presence of hydroxyl species generates competition with phosphates for interactions with the cationic sites of the sorbent. This results in lower sorption performances, particularly in most alkaline conditions (pH of 11.88). In this case, despite the presence of orthophosphate species, the concentration of hydroxyls hindered the interaction of the phosphates with the sorbent. Conversely, in acidic conditions (pH of 5.25), the removal is affected by the presence of protons in the solution, resulting in a slight decrease in performance.

**Figure 10:**
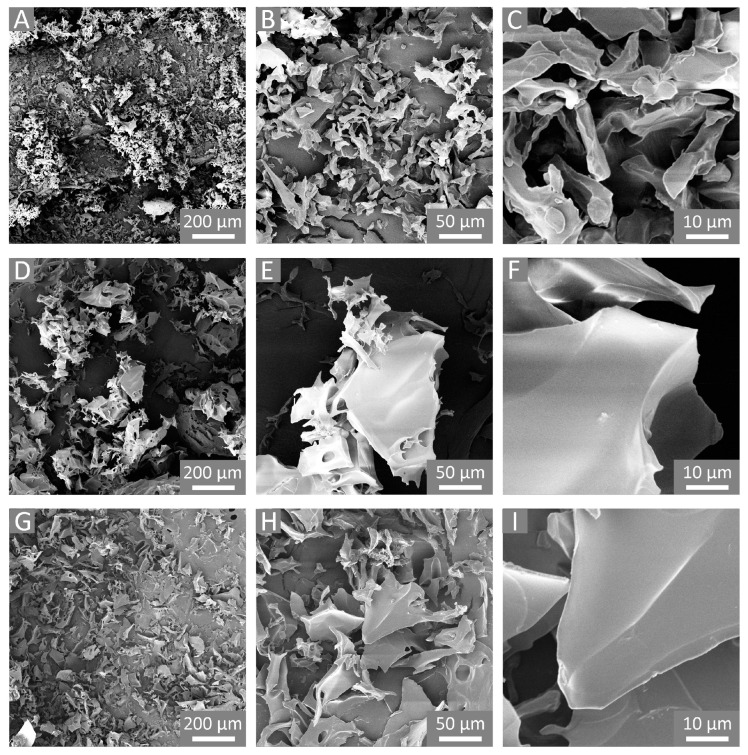
SEM images of (**A**–**C**) freeze-dried GLU_BDE after the nitrate removal test, (**D**–**F**) freeze-dried GLU_BDE after the sulfate removal test, and (**G**–**I**) freeze-dried GLU_BDE after the phosphate removal test.

**Figure 11:**
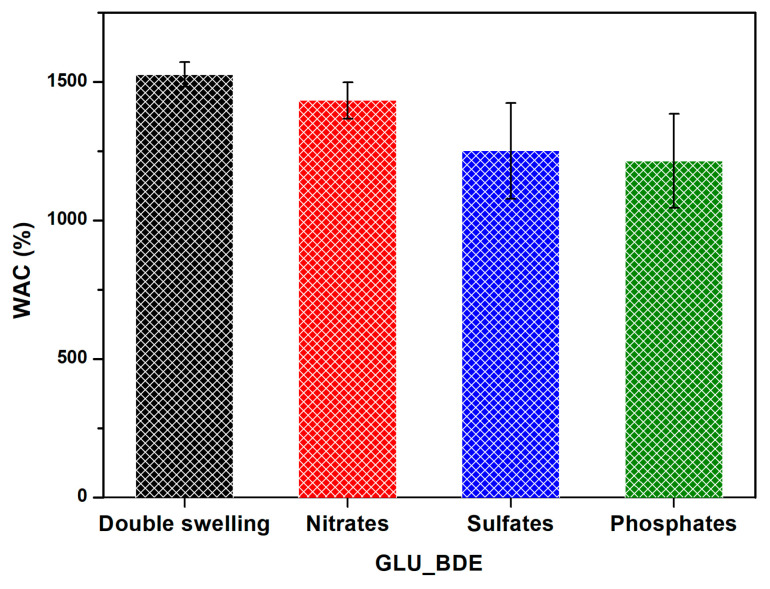
WAC of freeze-dried swollen GLU_BDE (Double swelling), freeze-dried GLU_BDE after the nitrate removal test (Nitrates), freeze-dried GLU_BDE after the sulfate removal test (Sulfates), and freeze-dried GLU_BDE after the phosphate removal test (Phosphates).

Eventually, GLU_BDE that had been freeze-dried after the removal tests was further used to investigate the interactions between the anions and the sorbent. For this reason, the WAC and the subsequent freeze-drying processes were repeated. Figure 10 confirms the presence of irregular and heterogeneous surfaces, as previously described, associated with the shrinking of gels from a swollen state to a dry one. The presence of nitrates, sulfates, and phosphates within the polymer matrix resulted in a decrease in the swelling properties of approximately 25% to 35%, as reported in Figure 11 and summarized in Table 1 with the results previously discussed.

## 3. Conclusions

Bio-derived cross-linked polymers were successfully obtained via a sustainable approach, exploiting commercial maltodextrin (Glucidex 2^®^, GLU2) as the building block. The amine-mediated epoxy ring-opening reactions of 1,4 butanediol diglycidyl ether (BDE) and trimethylolpropane triglycidyl ether (TTE) were exploited, using 1,4-diazabicyclo [2.2.2] octane to obtain the final polymer products, namely, GLU_BDE and GLU_TTE. The atom economy of the chosen synthetic approach allowed us to obtain high yields, while the polymer structures and morphology were studied via FTIR-ATR, TGA, DSC, XRD, SEM, elemental analysis, and zeta-potential measurements. Polydispersed amorphous cationic polymer granules were observed as a result. Subsequently, the obtained polymers were tested as novel bio-derived gel-forming systems by water absorption capacity measurements, cross-linking density determination via the Flory–Rehner theory, and rheology. Swelling properties ranging from 800% to 1500% and the viscoelastic behavior of cross-linked systems have been detected. Nitrate, sulfate, and phosphate removal tests were performed to evaluate the efficacy of the studied polymers as suitable sorbents for water remediation. GLU_TTE displayed the highest-performing system by reaching, from 100 mg/L solutions, a sorption percentage of 79.7 ± 4.0% and 94.6 ± 4.7% toward nitrates and sulfates, respectively, while total sorption regarding phosphates at pH 8.49 was observed. The percentage of removal was demonstrated to be higher at lower anion concentrations, while the grams of pollutant taken up per gram of sorbent showed higher levels from more concentrated starting solutions. Eventually, phosphate removal efficiency showed a dependency on the pH value and a maximum sorption percentage at pH 8.49. In this work, we demonstrated how the combination of maltodextrin, diglycidyl ether, triglycidyl ether, and amine represents a useful and sustainable approach for the one-step synthesis of cationic high-swelling cross-linked polymers, avoiding the use of organic solvents. In addition, their cationic features and good hydrophilicity were pivotal for the removal of nitrates, sulfates, and phosphates, demonstrating the need for these materials to be further studied as bio-derived sorbents for environmental applications.

## 4. Materials and Methods

### 4.1. Materials

Glucidex 2^®^ (GLU2) was provided by Roquette Frères (Lestrem, France), while 1,4-diazabicyclo [2.2.2] octane (DABCO), 1,4 butanediol diglycidyl ether (BDE), trimethylolpropane triglycidyl ether (TTE), potassium nitrate, sodium sulfate, sodium orthophosphate, sodium mono-hydrogen phosphate, and sodium di-hydrogen phosphate were purchased from Sigma-Aldrich (Darmstadt, Germany). GLU2 was dried in an oven at 75 °C up to a constant weight before use.

### 4.2. BDE Cross-Linked Polymer (GLU_BDE)

In a typical procedure, the GLU_BDE was synthesized by dissolving 3.5 g of GLU2 in 10 mL of 0.2 M sodium hydroxide aqueous solution, using a round-bottomed flask. Afterward, while keeping the solution under stirring, 0.25 g of DABCO was added. Eventually, 0.79 g of BDE was added, and the temperature was increased to 70 °C. The reaction was then allowed to continue for 90 min, obtaining a monolith block as the product. Subsequently, the product was crushed, recovered, and purified with deionized water to remove any non-reacted reagents. At the end of the purification step, the product was dried in an oven at 70 °C up to a constant weight and then ground with a mortar, thereby obtaining a powder. The expected chemical structure of the synthesized polymer is reported in Figure 1A.

### 4.3. TTE Cross-Linked Polymer (GLU_TTE)

In a typical procedure, the GLU_TTE was synthesized by dissolving 3.5 g of GLU2 in 10 mL of 0.2 M sodium hydroxide aqueous solution, using a round-bottomed flask. Afterward, while keeping the solution under stirring, 0.25 g of DABCO was added. Eventually, 1.35 g of TTE was added and the temperature was increased to 70 °C. The reaction was then allowed to continue for 90 min, obtaining a monolith block as the product. Subsequently, the product was crushed, recovered, and purified with deionized water, to remove any non-reacted reagents. At the end of the purification step, the product was dried in an oven at 70 °C up to a constant weight and ground with a mortar, thereby obtaining a powder. The expected chemical structure of the synthesized polymer is reported in Figure 1B.

### 4.4. Scanning Electron Microscopy (SEM)

The morphology of the samples was studied using SEM. The images were acquired with a Tescan VEGA 3 (Brno, Czech Republic) SEM using secondary electrons and 8 kV accelerating voltage. Before SEM characterization, the samples were ion-coated with 12 nm of gold, using a Vac Coat DSR1 sputter coater (London, UK).

### 4.5. Thermal Analyses

Thermogravimetric analysis (TGA) was carried out using a TA Instruments Discovery 550 TGA (New Castle, DE, USA) device from 50 °C to 700 °C, under nitrogen flow, with a heating rate of 10 °C/min. Differential scanning calorimetry (DSC) was carried out using a TA Instruments Q200 DSC (New Castle, DE, USA) device from 50 °C to 180 °C, under nitrogen flow, with a heating rate of 10 °C/min.

### 4.6. Fourier Transform Infrared Spectroscopy (FTIR)

A Perkin Elmer Spectrum 100 FT-IR Spectrometer (Waltham, MA, USA) equipped with a Universal ATR sampling accessory was used for FTIR-ATR (attenuated total reflection) characterization. All the spectra were collected in the wavenumber range of 650–4000 cm^−1^ at room temperature, with a resolution of 4 cm^−1^ and 8 scans/spectrum.

### 4.7. Powder X-ray Diffraction

Powder XRD patterns were recorded using a Panalytical X’Pert PRO diffractometer working in Bragg–Brentano geometry and equipped with a Cu-anode X-ray source (λ = 1.541 Å). Data were collected over an angular range from 3 to 70 ᵒ2θ, using a step size of 0.017 °2θ and a time per step of 74.93 s, in capillary configuration (capillary diameter = 0.8 mm).

### 4.8. Elemental Analysis

The chemical composition of the samples was studied using a Thermo Fisher FlashEA 1112 series elemental analyzer (Waltham, MA, USA).

### 4.9. Zeta Potential

A Malvern Zetasizer Nano–ZS (Malvern, UK) was used to measure the zeta potential. All the tests were performed using ultrapure water and were conducted at room temperature.

### 4.10. Water Absorption Capacity (WAC)

The water absorption capacity measurements were performed by adding 500 mg of dry powders (GLU_BDE and GLU_TTE) in a 15 mL test tube of deionized water, following a procedure described in a previous work [53]. The test tubes were sealed and kept at room temperature. After 20 h, the mixtures were centrifuged to divide into a layer of water-bound material and free unabsorbed water. After removing the supernatant, the weight of the swollen polymer was recorded. The WAC measurements were executed in triplicate for both GLU_TTE and GLU_BDE.

The swelling rate (%S) or the water absorption capacity (%WAC) was calculated using the following equation:

(1) $$WAC\left(\%\right)=\frac{m_{t}-m_{o}}{m_{o}}\times 100$$

where *m_t_* is the weight of the swollen sample and *m_o_* is the initial weight of the dry sample.

The swollen samples were subsequently freeze-dried.

### 4.11. Cross-Linking Density Determination Using Swelling Experiments

The water absorption capacity study permitted the calculation of the polymer volume fraction in the equilibrium-swollen polymer (ʋ_2m_), which is utilized to calculate the cross-linking density (ʋ) using the Flory–Rehner theory. The number of cross-links per unit volume in a polymer network is defined as cross-linking density, whereas the polymer volume fraction is related to the quantity of water that a polymer can incorporate.

The molecular weight between two cross-links (M_c_) is calculated using the Flory–Rehner equation as it is presented in the following equation:

(2) Mc=V1[(ʋ2m)^1/3−(2fʋ2m)]−[ln(1−ʋ2m)+ʋ2m+χ1(ʋ2m)2]

where χ_1_ is the Flory–Huggins solvent–polymer interaction parameter, *V*_1_ is the molar volume of water as a swelling agent, and *f* is the functionality of the cross-links. The relationship between M_c_ and ʋ is given by the following equation:

(3) $$M_{c}=\frac{\rho_{p}}{ʋ}$$

where *ρ_p_* is the polymer density. The density of the polymer is precisely determined using a calibrated pycnometer.

Cross-linking determination was performed according to the procedure previously described by our research group [53]. All the measurements were performed in triplicate.

### 4.12. Rheological Measurements

Rheological measurements were performed with a Rheometer TA Instruments Discovery HR 1 device by following the procedure described in our previous work [53]. The instrument was equipped with 20 mm diameter stainless steel plate geometry and Peltier plate temperature control. Frequency sweep measurement was performed from 100 to 0.2 rad/s, with 5 points per decade, and a stress amplitude of 2%. The amplitude sweep test was used to check the value of stress amplitude, guaranteeing the performance of the measurements within the linear viscoelastic region. The oscillatory shear mode was used to determine the shear modulus (G), in particular, the storage modulus (G′) and the loss modulus (G″) of the swollen polymers as a function of frequency (frequency sweep test) and as a function of shear strain (amplitude sweep test). The sample was placed between the upper parallel plate and a stationary surface, with a gap size of 2 mm. Roughened surface geometry, such as a crosshatched plate, was utilized to strengthen the contact between the geometry and the sample.

The fraction of elastically effective network chains is stipulated by the modulus measurements. The value of the plateau modulus G′_p_, measured by rheological measurements, is directly associated with the number of elastically effective chains per unit volume (ʋ_e_), as expressed in the following equation:

(4) $$G\prime p=(1-\frac{2}{f})\times {ʋ}_{e}\times RT$$

where ʋ_e_ is the molar number of elastically effective network chains per unit volume estimated in mol m^−3^, R is the universal gas constant (8.314 J mol^−1^ K^−1^), T is the temperature, and *f* is the functionality as formerly defined.

### 4.13. Sorption Tests

The sorption tests were performed using 1 g of cationic sorbent for 100 mL of pollutant solution. The sorption performance regarding nitrates and sulfates was tested at different concentrations. Potassium nitrate and sodium sulfate were used to prepare 100, 200, 300, 400, 500, and 1000 mg/L solutions of each anion. In the case of phosphates, the sorption was studied at different pH values. Sodium orthophosphate, sodium mono-hydrogen phosphate, and sodium di-hydrogen phosphate were used to obtain phosphate-containing water solutions characterized by pH values of 11.88, 8.49, 7.04, and 5.25, produced while keeping the concentration of phosphorous constant at 32 mg/L. Concentrations of pollutants lower than 400 mg/L were chosen to simulate the treatment of different grades of polluted waters, in compliance with the Italian Environmental Law limits (D. Lgs. 152/06—Appendix 3), while concentrations of higher than 400 mg/L were intended to evaluate the saturation grade of each sorbent.

Each sorption test was carried out by stirring the sorbent dispersion for 24 h at room temperature using an Enrico Bruno (Turin, Italy) orbital shaker. Afterwards, the solution was filtered and analyzed with the ion chromatography technique using a MetrOhm (Herisau, Switzerland) 883 Basic IC plus, with 1 mM of NaHCO_3_ and 3.2 mM of Na_2_CO_3_ as the eluent, and MetrOhm (Herisau, Switzerland) Metrosep A Supp 5 250/4.0, 250 × 0.4 mm as the column. The effectiveness of the polymers in sorbing the contaminants from water was expressed as the percentage of sorption (*Sor*_(%)_):

(5) $${Sor}_{\left(\%\right)}=\left(1-\frac{{Conc\;t}_{x}}{{Conc\;t}_{0}}\right)\times 100.$$

In parallel, the sorption capacity was determined, as represented by the number of milligrams of anions removed per gram of sorbent (*Sor*_(*mg*/*g*)_):

(6) $${Sor}_{\left(mg/g\right)}=\left({Conc\;t}_{0}-{Conc\;t}_{x}\right)\times \frac{V}{m}$$

where *Conc t*_0_ (mg/L) represents the initial concentration of the pollutant, *Conc t_x_* (mg/L) is the concentration of anions after each time interval, *V* (L) is the volume of the pollutant solution, and *m* (g) is the mass of sorbent used. Three replicates were carried out for each test.

## Figures and Tables

**Table 1:** Summary of the reported results.

Test Performed	Sorbent	
	GLU_BDE	GLU_TTE
T_onset_ (°C)	271	264
N wt.%	0.8	1.1
Zeta potential (mV)	10.4 ± 1.2	13.8 ± 1.7
WAC (%)	1293 ± 13	755 ± 5
WAC (%) double swelling	1560 ± 46	878 ± 56
WAC (%) after NO_3_^−^ sorption	1433 ± 66	/
WAC (%) after SO_4_^2−^ sorption	1251 ± 173	/
WAC (%) after PO_4_^3−^ sorption	1215 ± 170	/
G′ (Pa)	2435	1285
G″ (Pa)	899	691
υ^FR^ (mol/cm^3^)	1.72 × 10^−5^ ± 6.55 × 10^−7^	5.64 × 10^−5^ ± 1.26 × 10^−6^
υ_e_^R^ (mol/cm^3^)	2.38 × 10^−6^ ± 9.68 × 10^−7^	2.83 × 10^−6^ ± 1.86 × 10^−6^
M_c_ (g/mol)	58,212 ± 2322	17,724 ± 385
Sor_(%)_ NO_3_^−^, 100 mg/L	62.9 ± 3.1	79.7 ± 4.0
Sor_(mg/g)_ NO_3_^−^, 100 mg/L	6.3 ± 3.1	8.0 ± 4.0
Sor_(%)_ NO_3_^−^, 200 mg/L	54.7 ± 2.2	65.9 ± 2.6
Sor_(mg/g)_ NO_3_^−^, 200 mg/L	10.9 ± 2.2	13.2 ± 2.6
Sor_(%)_ NO_3_^−^, 300 mg/L	41.1 ± 1.4	57.4 ± 1.9
Sor_(mg/g)_ NO_3_^−^, 300 mg/L	12.3 ± 1.4	17.2 ± 1.9
Sor_(%)_ NO_3_^−^, 400 mg/L	29.0 ± 0.7	48.6 ± 1.2
Sor_(mg/g)_ NO_3_^−^, 400 mg/L	11.6 ± 0.7	19.4 ± 1.2
Sor_(%)_ NO_3_^−^, 500 mg/L	21.4 ± 0.4	43.2 ± 0.9
Sor_(mg/g)_ NO_3_^−^, 500 mg/L	10.7 ± 0.4	21.6 ± 0.9
Sor_(%)_ NO_3_^−^, 1000 mg/L	18.0 ± 0.3	38.0 ± 0.6
Sor_(mg/g)_ NO_3_^−^, 1000 mg/L	18.0 ± 0.3	38.0 ± 0.6
Sor_(%)_ SO_4_^2−^, 100 mg/L	64.2 ± 3.2	94.6 ± 4.7
Sor_(mg/g)_ SO_4_^2−^, 100 mg/L	6.4 ± 3.2	9.4 ± 4.7
Sor_(%)_SO_4_^2−^, 200 mg/L	51.0 ± 2.0	89.9 ± 3.6
Sor_(mg/g)_ SO_4_^2−^, 200 mg/L	10.2 ± 2.0	18.0 ± 3.6
Sor_(%)_ SO_4_^2−^, 300 mg/L	41.6 ± 1.4	77.2 ± 2.6
Sor_(mg/g)_ SO_4_^2−^, 300 mg/L	12.5 ± 1.4	23.2 ± 2.6
Sor_(%)_ SO_4_^2−^, 400 mg/L	31.8 ± 0.8	61.4 ± 1.5
Sor_(mg/g)_ SO_4_^2−^, 400 mg/L	12.7 ± 0.8	24.6 ± 1.5
Sor_(%)_ SO_4_^2−^, 500 mg/L	25.0 ± 0.5	51.6 ± 1.0
Sor_(mg/g)_ SO_4_^2−^, 500 mg/L	12.5 ± 0.5	25.8 ± 1.0
Sor_(%)_ SO_4_^2−^, 1000 mg/L	15.4 ± 0.3	29.4 ± 0.5
Sor_(mg/g)_ SO_4_^2−^, 1000 mg/L	15.4 ± 0.3	29.4 ± 0.5
Sor_(%)_ PO_4_^3−^, pH 11.88	51.2 ± 1.6	93.8 ± 2.4
Sor_(mg/g)_ PO_4_^3−^, pH 11.88	16.7 ± 1.6	30.6 ± 2.4
Sor_(%)_ PO_4_^3−^, pH 8.49	83.5 ± 1.3	99.0 ± 0.2
Sor_(mg/g)_ PO_4_^3−^, pH 8.49	26.9 ± 1.3	31.9 ± 0.2
Sor_(%)_ PO_4_^3−^, pH 7.04	84.6 ± 3.2	91.1 ± 3.3
Sor_(mg/g)_ PO_4_^3−^, pH 7.04	27.0 ± 3.2	29.1 ± 3.3
Sor_(%)_ PO_4_^3−^, pH 5.25	79.5 ± 4.1	92.2 ± 2.9
Sor_(mg/g)_ PO_4_^3−^, pH 5.25	25.5 ± 4.1	29.6 ± 2.9

## Data Availability

The data presented in this study are openly available in the article.
